# Subject Gaps Revisited: Complement Clauses and Complementizer-Trace Effects

**DOI:** 10.3389/fpsyg.2021.658364

**Published:** 2021-05-19

**Authors:** Rebecca Tollan, Bilge Palaz

**Affiliations:** Department of Linguistics and Cognitive Science, University of Delaware, Newark, DE, United States

**Keywords:** long-distance dependencies, filled gap effects, subjecthood, embedded clauses, complementizers

## Abstract

This study investigates how filler-gap dependencies associated with subject position are formed in online sentence comprehension. Since Crain and Fodor (1985), “filled-gap” studies have provided evidence that the parser actively seeks to associate a *wh-filler* with a gap in direct object position of a sentence wherever possible; the evidence that this same process applies for subject position, is, however, more limited (Stowe, 1986; Lee, 2004). We examine the processing of complement clauses, finding that *wh* dependency formation is actively attempted at embedded subject position (e.g., *Kate* in *Who did Lucy think Kate could drive us home to?*), unless, however, the embedded clause contains a complementizer (e.g., *Who did Lucy think that Kate* … *.?*). The absence of the dependency formation in the latter case demonstrates that the complementizer-trace effect (cf., *^∗^Who did Lucy think that could drive us home to mom?*; Perlmutter, 1968) is, like syntactic island constraints (Ross, 1967; Keshev and Meltzer-Asscher, 2017), immediately operative in online structure building.

## Introduction

Constructions such as *wh* questions involve filler-gap dependencies, in which a displaced *wh-phrase* (the “filler”) appears in a place in the sentence which is not the location in which it receives its thematic interpretation (the “gap”). For example, the embedded *wh* interrogative clause in (1) involves a dependency between *who* and the post-verbal site (after *drive*) such that *who* is analyzed as a direct object.

(1) Alice asked [**who** Kate could drive __ home].

In real-time sentence processing, the parser aims to associate a *wh-filler* with a gap as quickly as possible, as per the “Active Filler Strategy” (Frazier, 1978); that is, dependency formation is attempted at the first potential site encountered. In (2), for example, a filler-gap dependency is expected following the verb *drive*, which is the first potential gap site. When *grandad* is encountered, it becomes apparent that the *wh* dependency cannot be formed here. This scenario causes momentary processing difficulty, as the filler must be held for longer in working memory, until the eventual gap (after *to*) is located.

(2) Alice asked [**who** Kate could drive grandad home to __].

In reading-time studies (Crain and Fodor, 1985; et seq.), this processing difficulty is indicated by a slowdown in reading time at the direct object site, *grandad*, relative to the same site in a baseline non-*wh* construction, such as the embedded polar interrogative in (3).

(3) Alice asked [if Lucy could drive grandad home to mom].

Building on Crain and Fodor (1985), Stowe (1986) conducted a reading-time study in which reading times in sentences containing an embedded *wh* question like (4a) were compared with embedded polar interrogatives like (4b). Indeed, Stowe found a robust filled-gap effect at the direct object position (i.e., *us*), providing evidence that the parser had attempted to form a *wh* dependency after *bring* in (4a).

(4) a. My brother wanted to know [who Ruth will bring *us* home to __ at Christmas].
  b. My brother wanted to know [if Ruth will bring *us* home to mom at Christmas].
  (Stowe, 1986: 234)

Curiously, however, Stowe did not find any indication of a filled gap in *subject* position in (4). That is, there was no slowdown at *Ruth* in (4a) relative to in (4b); Stowe concluded that subject gaps are not treated in the same manner as object gaps, at least insofar as failure to locate an expected gap in subject position does not cause the same processing difficulty as is found for object position. Stowe reasoned that gaps in subject position are treated differently by the parser as compared to gaps in object position, and hypothesized that this asymmetry could be due to either (i) a *wh-phrase* being treated by default as a subject, such that it is only when an alternative subject is identified that the search for a gap in an alternative location actively begins, or (ii) recovery from a subject filled gap being less burdensome than from an object filled gap. According to Stowe, such a contrast might be due to the word order of English, which is S(ubject)-V(erb)-O(bject). This means that when an object or object gap is encountered, a complete semantic proposition can be formed. In contrast, encountering a subject or subject gap does not lead to this same outcome, because the verb and object have yet to be parsed. Thus, recovering from an object filled gap would be burdensome because the parser is simultaneously also computing a semantic proposition.

Others have proposed that the reason for the absence of a subject filled gap effect in (4) is one of relative *timing* (Clifton and Frazier, 1989; Lee, 2004; Wagers and Pendleton, 2016): because, in English, the subject gap occurs immediately after the filler, the lack of subject filled gap effect could be an indication of the parser having had insufficient time to create an expectation of a gap in the first place. As evidence in support of this hypothesis, Lee (2004) and Wagers and Pendleton (2016) found that, when adverbial material is inserted between the filler and the subject, such as “after the party was over,” as in the pair of sentences in (5), then a filled gap effect at subject position emerged.

(5) a. Alice asked [who, **after the party was over**, *Kate* could drive grandad home to __].
  b. Alice asked [if, **after the party was over**, *Kate* could drive grandad home to mom].

The timing hypothesis makes a clear prediction: if the absence of a subject filled gap effect in (4a) is due to the adjacency of the filler and the subject position, as opposed to being due to any intrinsic properties of grammatical subjects, then only the *highest* subject position – namely, that which is closest to the filler – should be affected. This means that a subject filled gap effect *is* expected to emerge in subject positions of complement clauses, such as in those in (6). That is, processing difficulty is predicted to occur at *Kate* in (6a) relative to (6b).

(6) a. Alice asked [who Lucy thought [*Kate* could drive grandad home to __]].
  b. Alice asked [if Lucy thought [*Kate* could drive grandad home to mom]].

The study of such types of complement clauses connects with a second line of inquiry which has received a lot of attention in sentence processing literature: does the parser actively attempt gap formation at positions in which a *noun phrase* is grammatically licensed, but at which a *gap* is not? To this end, we exploit the fact that the pair of sentences in (6) allows for a further manipulation directly relevant to this question: a subject dependency can only be formed in the absence of an overt complementizer, as in (7a). When the complement clause contains the overt complementizer *that*, then a subject dependency is ungrammatical, as in (7b). This constraint is well known as a *complementizer-trace* effect (Perlmutter, 1968).

(7) a. Alice asked [who Lucy thought [*__* could drive grandad home to mom]].
  b. ^∗^Alice asked [who Lucy thought [**that** __ could drive grandad home to mom]].

There is currently no consensus as to why complementizer-trace effects arise (see Pesetsky, 2017, for an overview of various syntactic and prosodic accounts). One recent and prevalent proposal in generative syntactic literature reasons that the ill-formedness of constructions like (7b) is due to a syntactic constraint known as *anti-locality* (Abels, 2003; Schneider-Zioga, 2007; Erlewine, 2016), which means that *wh*- or “A-bar movement” dependencies formed in natural language cannot be *too* short. Importantly, (7b) – but not (7a) – is argued to involve an intermediate dependency (Erlewine, 2020), whereby the *wh*-phrase *who* is intermittently re-activated and re-stored in working memory immediately prior to the complementizer *that*, and that the dependency between this intermediate site and the eventual post-*that* gap site is of insufficient length. Here, we note a connection between the formal theory of anti-locality and theories of active gap filling which appeal to *timing* to account for the absence of subject filled-gap effects in sentences like (4a). That is, if timing is a critical issue in active gap filling, then this might pave the way for explaining *why* natural language exhibits any such “anti-locality” constraints at all, given that, conversely, it is usually *locality* constraints (Rizzi, 1990) – or requirements for structural proximity – that are invoked to account for a vast range of other morphosyntactic phenomena.

In terms of our present study, we ask whether the complementizer-trace effect in (7b) is active in online filler-gap dependency formation. If it is, then no processing difficulty should be observed at *Kate* in (8a) relative to (8b), because the parser would not anticipate a subject gap following a complementizer.

(8) a. Alice asked [who Lucy thought [**that** *Kate* could drive grandad home to]].
  b. Alice asked [if Lucy thought [**that** *Kate* could drive grandad home to mom]].

The question of whether the parser actively avoids positing gaps in unlicensed locations has typically been investigated from the perspective of *syntactic islands* (Ross, 1967); that is, stretches of structure – such as that which is enclosed in square brackets in (9) – in which *wh* dependencies cannot be formed. For example, the ungrammaticality of a *wh* question such as (9a), in which a subject gap is posited inside of a clausal subject, entails that no active gap formation attempt should take place, and thus, no processing difficulty should be incurred when this position is filled, as in (9b) with *Kate.*

(9) a. ^∗^Who did [the fact that __ could drive grandad home to mom] surprise Lucy?
  b. Who did [the fact that *Kate* could drive grandad home to mom] surprise __?

As is characteristic of islands (and hence the term), no gap can be posited within a clausal subject whatsoever, regardless of position within the clausal subject. Instead, ill-formedness arises due to the syntactic environment in which the gap is situated. Sentence processing research has reached a consensus that the parser actively *avoids* positing gaps inside islands (see, e.g., Keshev and Meltzer-Asscher, 2017 for a recent overview). This has led to hypotheses that the search for a gap site, at which to form the required *wh* dependency in questions like in (9b), is intrinsically paused until the structure comprising the island has elapsed (i.e., until after *mom*). Accordingly, processing models have posited that the gap searching mechanism is “suppressed” while within the boundaries of the island (e.g., Traxler and Pickering, 1996).

We approach this issue from an alternative angle: does the parser posit gaps in positions that are unlicensed not by virtue of the syntactic environment alone, but by virtue of the grammatical function (e.g., subject, object) of the gap itself? Unlike the clausal subjects in (8) – (9), *that*-complement clauses in (6) – (7) are *not* islands for dependency formation: an object gap is permitted in a *that*-complement clause, as in (10).

(10) Alice asked [who Lucy thought [**that** Kate could drive __ home to mom]] (cf., 7b).

This means that the ill-formedness of (7b) is not a direct artifact of the environment of the gap but arises from its interaction with grammatical function; that is, it is the fact that the gap is in *subject* position which is relevant (cf., Stowe, 1986).

The current study therefore investigates two questions. First, we ask whether filled-gap effects can be detected at subject positions in embedded complement clauses as in (6). Answering this first component contributes to discussion pertaining to the nature of subject gaps: are they treated differently from object gaps by virtue of (i) an inherent property of subjecthood or (ii) timing? Second, we examine whether the complementizer-trace effect (cf., 7) pertaining to embedded clauses prohibits online subject dependency creation, or alternatively whether dependency formation is attempted because the environment in which the gap is situated is not impermeable to gaps (cf., 10), and therefore, the parser might not intrinsically pause its active gap search.

## Method

We administered a self-paced reading task, employing a 2 × 2 within-subjects design, in which question type (*wh*, polar) was crossed with complementizer type (null, *that*). Because sentences with multiple embeddings have been shown to incur extra processing difficulty (e.g., Babyonyshev and Gibson, 1999; Warren and Gibson, 2002), we were concerned that reading multiple sentences with double embedded clauses, as in (6–8), could make the experiment overly burdensome for participants; therefore, we opted instead to use matrix *wh* questions, as in (9). The resulting four conditions for our study are shown in Table 1.

**TABLE 1:** The four conditions used in the study: wh-null, polar-null, wh-that, polar-that.

Question type	Complementizer type	
	*null*	*that*
*wh*	Which family member did Lucy think Kate could drive grandad home to?	Which family member did Lucy think **that** Kate could drive grandad home to?
*polar*	Did Lucy think Kate could drive grandad home to mom?	Did Lucy think **that** Kate could drive grandad home to mom?

Comparing reading times at embedded subject *Kate* in *wh*-null with polar-null establishes whether a filled-gap effect arises at a (non-matrix) subject position. Comparing *Kate* in *wh-that* with polar-*that* establishes whether the complementizer-trace effect is active in online dependency formation.

However, one possible scenario is not supported by analyzing reading times for subject position (i.e., *Kate*) alone: if there is *no* subject filled-gap effect, whether in null or *that* clauses, then the outcome is a non-interpretable null result. Thus, we ensured that the questions also included an overt embedded object (i.e., *grandad* in Table 1), which would be expected to trigger a filled-gap effect in object position (Crain and Fodor, 1985; Stowe, 1986). In the event of a null result for subject position, detection of a filled-gap effect in object position would help to support an interpretation of such a null result as being due to the *absence* of the effect itself, rather than due to filled gap effects being undetectable in the context of our specific experimental set-up. This then prompted one final adjustment: in the polar conditions, the appearance of an embedded object results in a potentially well-formed end-of-sentence [i.e., Did Lucy think (that) Kate could drive grandad?], which would be expected to give rise to a sentence-final wrap-up effect (Just and Carpenter, 1980); that is, a reading time slowdown at *grandad* in the polar condition. Thus, we decided to use complex *which NP* fillers such as *which family member*, as previous reading-time studies have revealed that they give rise to larger filled-gap slowdowns relative to simplex *who* fillers (Tollan and Heller, 2016).

### Materials

Sixteen item sets following the format shown in Table 1 were created and rotated across four lists following a modified Latin-square design: each participant saw four items in each of the four conditions. To ensure that the embedded clausal subject could not be mis-parsed as a direct object of the matrix verb (cf., Trueswell and Kim, 1998), we used only matrix verbs which are incompatible with human direct objects (*think, decide, say, realize, insist*). Each item was preceded by a context story. The story which the item set in Table 1 was coupled with is given in (11) (*that* was included in the context story for *that* conditions, and not for null conditions).

(11) *Lucy was planning the Christmas holiday season for herself and her family. Everyone would gather at mom’s house. She thought (that) Alice could drive grandma and Kate could drive grandad.*

Importantly, (11) licenses both an embedded subject question (i.e., *Which family member did Lucy think __ could drive grandad/grandma home?*) and an embedded object question (i.e., *Which family member did Lucy think Kate/Alice could drive __ home?*), as well as the eventual prepositional object question (e.g., *Which family member did Lucy think Kate could drive grandad home to?*).

We also included 22 fillers contexts + questions. Because the correct answer to the critical polar questions was always “yes,” we included an additional eight polar questions for which the correct answer was “no.” To prevent participants from coming to expect that questions would not end with a verb, we included eight *which NP* (matrix) object questions. We also included six matrix prepositional object questions. Each participant thus saw 38 items, presented in a pseudo-random order (no two adjacent items were critical items).

### Participants

Thirty-four native speakers of English, recruited from the University of Delaware community, participated in exchange for $5 or one undergraduate course credit.

### Procedure

We used the online software Ibex Farm (Drummond, 2013). Participants were asked to read each story and answer the corresponding question. The context story was presented in full. The question was then presented in a non-cumulative word-by-word self-paced fashion. Participants then saw a multiple-choice answer set (*wh* questions: three possible answers, *polar* questions: *yes* or *no*); no feedback was given. Three practice trials were included at the beginning. The task took approximately 30 min to complete.

## Results

Two participants were excluded from analysis as their mean comprehension accuracy was below 75%. We report data from the remaining 32 participants: thus, 512 critical trials. Of these, the mean accuracy for comprehension questions was 82.4%; trials which generated incorrect responses (*n* = 90) were excluded from further analysis^1^. One additional trial was then excluded because it contained an unexpectedly long single-word reading time (37 s). This left us with 421 trials for analysis.

**FIGURE 1:**
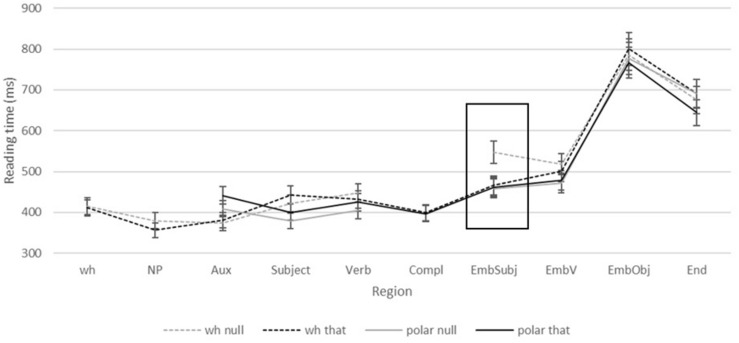
Mean reading times for each region of the wh question, by condition. The primary region of interest (the embedded subject position) is indicated with a box. Error bars show 95% Confidence Intervals.

We then analyzed the time course of reading for the *wh* question. First, for each region, we dealt with outliers for by calculating the mean reading time and standard deviation for each condition and replaced trials which were 2SD above or below the mean for that condition by the condition mean. This procedure affected approximately 0.11% of the data at each region. Following this, we computed mean reading times by region of the *wh* question, as shown in Figure 1.

For each region, we analyzed the reading times by fitting a mixed-effects linear regression with crossed random effects for participants and items (Baayen et al., 2008), using the *lme4* package (R 4.0.2: Bates et al., 2015). Each model included the maximal random effect structure that would allow for convergence.

We begin by focusing on the critical region, the embedded subject position (i.e., *Kate* in Table 1). Here, the 2 × 2 analysis revealed a main effect of question type, with reading times for *wh* questions significantly longer than those for polar questions (507 vs. 461 ms; β = 87.57, *SE* = 29.69, *t* = 2.95 *p* = 0.008), no main effect of complementizer type – meaning that null clauses and *that* clauses did not significantly differ, overall (*p* > 0.8) – and a significant interaction (β = −87.24, *SE* = 34.96, *t* = −2.5, *p* < 0.018). Importantly, planned comparisons showed that reading times for *wh* questions were significantly longer than for polar questions in the null conditions (547 vs. 459 ms; β = 87.67, *SE* = 29.69, *t* = 2.95, *p* = 0.008); this indicates a filled-gap effect in embedded subject position in a null complement clause. This same effect is, however, absent in a *that* complement clause: reading times for *wh-that* did not differ from *polar-that* (466 vs. 452 ms; β = *0.33*; *SE* = 22.08, *t* = 0.015, *p* = 0.99), indicating no hint of a subject filled gap effect when the complement clause contains an overt complementizer, and therefore, that complementizer-trace effects are actively adhered to in online sentence processing.

The subject filled gap effect in the *wh* null condition persisted into the embedded verb region (immediately following the embedded subject), with *wh* null being significantly slower than polar null (518 vs. 472 ms; *t* = 2.92; *p* = 0.004), but there was no difference between *wh* that and polar *that* (500 vs. 479 ms; *t* = 1.27; *p* = 0.21). Curiously, there were no significant main effects or interactions at the embedded object position (grand mean RT = 782 ms; all *p*s > 0.7), and neither planned comparison showed statistical significance (both *p*s > 0.57). This indicates no observable filled-gap effects at object position; surprisingly, this null result for object position does *not* replicate the well-known object filled gap effect found in numerous prior studies, including Stowe (1986). We hypothesize that this discrepancy may be due to the type of questions used in our study, which differs from that of Stowe (among others): our study involved *matrix* questions, which participants were required to answer directly by selecting from two or more options, in which object filled gap effects may have been supplanted by end-of-sentence wrap-up effects (Just and Carpenter, 1980). Conversely, *embedded* questions, such as those used in Stowe (1986) (see again the pair of sentences in 4) do not constitute a direct request for information. Participants may be less sensitive to end-of-sentence effects when the relevant question does not require a direct answer, as compared with the matrix questions used in our materials, which do. Because end-of-sentence effects are not relevant to our main research questions pertaining to subject gaps, we set this matter aside for the remainder of our discussion but raise it as a topic for future consideration.

Elsewhere in the sentence, we observe main effects of *wh* type at the matrix auxiliary position, where *wh* questions were faster than polar questions (378 vs. 426 ms; *t* = −2.89; *p* = 0.007), whereas wh questions were slower than polar questions at both the matrix subject position (433 vs. 390 ms; *t* = 2.68; *p* = 0.0099) and verb position (440 vs. 414 ms; *t* = 3.12; *p* = 0.0042). There were no significant effects of complementizer type, however, at any region besides the embedded subject and verb positions (all *p*s > 0.1)^2^.

## Discussion

This study found evidence for active gap formation in subject position of an embedded complement clause, providing indication that the absence of filled gap effect in matrix subject position, as in Stowe (1986), is due to adjacency of filler and matrix subject gap site (Clifton and Frazier, 1989; Lee, 2004; Wagers and Pendleton, 2016), rather than any inherent properties of subject position^3^. Thus, we provide further support for a view in which location of a subject gap proceeds in a manner analogous to that of an object gap, once the parser has been afforded the necessary time to initiate a gap search. Our conclusion concerning the importance of timing in filler-gap dependency formation connects with generative syntactic theory, within which several cross-linguistic filler-gap (a.k.a. “A-bar movement”) phenomena have led to proposing *anti-locality* theory (Abels, 2003; Schneider-Zioga, 2007; Erlewine, 2016). We might understand such a constraint as, in fact, being grounded in principles of filler-gap dependency processing: if formation of an overly short *wh* dependency chain is not actively attempted in real-time sentence comprehension, then it follows that such types of dependencies would not constitute part of the syntax of natural language (Hawkins, 2004).

The embedded subject filled gap effect is, however, absent when the complement clause contains the overt complementizer *that*, indicating that the complementizer-trace effect which gives rise to ill-formed constructions such as ^∗^*Who did Lucy think that __ could drive us home to mom?* is actively adhered to in online gap location processes. This indicates that suppression of an active search for a gap is guided directly by whatever linguistic constraints govern well-formedness of a dependency, and not by sole virtue of the structural environment in which the dependency is located. Applying this to findings from the literature on the processing islands, this provides preliminary support for a view in which adherence to island constraints is not an artifact of the parser simply “stalling” the gap search process until outside of the island (e.g., Traxler and Pickering, 1996), but of independent evaluation of whether each gap site in turn would result in a well-formed *wh* dependency.

## Data Availability Statement

The raw data supporting the conclusions of this article will be made available by the authors, without undue reservation.

## Ethics Statement

The studies involving human participants were reviewed and approved by the University of Delaware. The patients/participants provided their written informed consent to participate in this study.

## Author Contributions

RT designed and made materials for the study, conducted the statistical analyses of the data from the study, and wrote the manuscript. BP tested participants, processed and analyzed the data from the study, and reviewed the manuscript. Both authors contributed to the article and approved the submitted version.

## Conflict of Interest

The authors declare that the research was conducted in the absence of any commercial or financial relationships that could be construed as a potential conflict of interest.

## Footnotes

1 A mixed-effects logistic regression model fitted to the accuracy data revealed no significant main effects or interaction (all *p*s > 0.2).

2 One limitation of our study concerns statistical power: A *post hoc* power analysis of our model fitted to RTs at embedded subject position, run using the SIMR package (Green and MacLeod, 2016; *nsim* = 1000), estimated our sample to have 65.4% power. Low power means that any non-significant results are not possible to interpret; in particular, we cannot make a strong claim that an effect is *absent*, merely that we failed to find evidence for an effect.

3 One alternative possibility is that the context story itself created an expectation for an embedded subject *wh* question, such that the filled gap effect in the null *wh* condition reflects disruption of prior expectation rather than active gap-filling. If this is the case, then we might also expect a difference between the *wh*-that condition and the polar-that conditions at the complementizer position, because the presence of a complementizer in the *wh* condition would signal that the embedded question cannot be a subject *wh* question (in view of the observation that the parser actively attends to complementizer-trace effects). No indication of such an effect was found, however.
